# The Advantages of New Multimodality Imaging in Choosing the Optimal Management Strategy for Patients with Hypertrophic Cardiomyopathy

**DOI:** 10.3390/diagnostics10090719

**Published:** 2020-09-19

**Authors:** Larisa Anghel, Cristian Stătescu, Ionela-Lăcrămioara Șerban, Minela Aida Mărănducă, Doina Butcovan, Alexandra Clement, Mădălina Bostan, Radu Sascău

**Affiliations:** 1 Internal Medicine Department, “Grigore T. Popa” University of Medicine and Pharmacy, 700503 Iași, Romania; larisa.anghel@umfiasi.ro (L.A.); madalina_farima@yahoo.com (M.B.); radu.sascau@gmail.com (R.S.); 2 Cardiology Department, Cardiovascular Diseases Institute “Prof. Dr. George I.M.Georgescu”, 700503 Iași, Romania; doinabutcovan@outlook.com (D.B.); alexandram.clement@gmail.com (A.C.); 3 Physiology Department, “Grigore T. Popa” University of Medicine and Pharmacy, 700503 Iași, Romania; ionela.serban@umfiasi.ro (I.-L.Ș.); minela.maranduca@umfiasi.ro (M.A.M.)

**Keywords:** hypertrophic cardiomyopathy, cardiac magnetic resonance, myocardial strain, computed tomography angiography, cardiac nuclear imaging

## Abstract

In recent years, significant advances have been made in the diagnosis and therapeutic management of hypertrophic cardiomyopathy (HCM) patients, which has led to an important improvement in their longevity and quality of life. The use of multimodality imaging has an essential role in the diagnosis, assessing the regional distribution and severity of the disease, with important prognostic implications. At the same time, imaging contributes to the identification of optimal treatment for patients with hypertrophic cardiomyopathy, whether it is pharmaceutical, interventional or surgical treatment. Novel pharmacotherapies (like myosin inhibitors), minimally invasive procedures (such as transcatheter mitral valve repair, high-intensity focused ultrasound or radiofrequency ablation) and gene-directed approaches, may soon become alternatives for HCM patients. However, there are only few data on the early diagnosis of patients with HCM, in order to initiate treatment as soon as possible, to reduce the risk of sudden cardiac death (SCD). The aim of our review is to highlight the advantages of contemporary imaging in choosing the optimal management strategies for HCM patients, considering the novel therapies which are currently applied or studied for these patients.

## 1. Introduction

Hypertrophic cardiomyopathy (HCM) is the most frequent genetic heart disease, usually caused by mutations in genes that encode sarcomeric proteins [1]. It is defined by unexplained progressive left ventricular hypertrophy (LVH), systolic and diastolic ventricular dysfunction, arrhythmia that can cause sudden cardiac death (SCD) and histopathologic modifications, such as myofibrillar disarray and myocardial fibrosis [2,3,4,5]. In the adult population, the prevalence of hypertrophic cardiomyopathy is estimated at 0.16% to 0.29%, with a higher prevalence in older patients, considering the age-dependent expression of gene mutations [6,7,8,9]. The disease was first described in 1958 [10] and was regarded as a pathology without treatment that could favorably influence the prognosis, so it was considered the most common cause of SCD in young people [11]. The clinical presentation, treatment and prognosis of patients with HCM is very variable, and this heterogeneity has led to controversy in choosing the optimal management strategy for these patients. The main symptoms for patients with HCM are dyspnea, chest pain, palpitations, a decreased exercise tolerance or syncope. There is no correlation between symptoms and signs of HCM and the severity of the left ventricular hypertrophy or left ventricular outflow tract (LVOT) obstruction. Additionally, a large number of young patients with HCM are asymptomatic or minimally symptomatic their whole lives [12]. Sudden cardiac death, although not frequent (0.31 to 0.39 per HCM person years), still represents the most devastating manifestation of hypertrophic cardiomyopathy [13], thus identifying patients at increased risk of SCD is extremely important. The European Society of Cardiology Guideline defines HCM in adults by a wall thickness ≥15 mm in one or more left ventricular myocardial segments, LVH that is not caused only by loading conditions, such as valvopathies and hypertension [2]. Even though in most cases the ventricular hypertrophy affects the interventricular septum and is severe and asymmetric, this is not a specific modification, as it can also be found in patients with isolated interventricular septum hypertrophy or in those with hypertensive cardiomyopathy [14]. The presence of myocardial hypertrophy in patients with aortic stenosis or arterial hypertension is a challenge for the clinician, in order to make the differentiation of primary hypertrophy from secondary hypertrophy. The use of multimodality imaging helps to make a correct differential diagnosis and also contributes to a precise characterization of the left ventricle wall and chamber, mitral valve and myocardial tissue. So, imaging tests have an essential role in the diagnosis, assessing the regional distribution and severity of the disease, with important prognostic implications [15]. At the same time, imaging contributes to the identification of optimal treatment for patients with hypertrophic cardiomyopathy, whether it is pharmaceutical, interventional or surgical treatment. However, there are only few data on the early diagnosis of patients with HCM, in order to initiate treatment as soon as possible, to reduce the risk of SCD. The aim of our review is to highlight the advantages of contemporary imaging in choosing the optimal management strategies for patients with hypertrophic cardiomyopathy, considering the novel therapies which are currently applied or studied for these patients.

## 2. Echocardiography

Transthoracic echocardiography remains the cornerstone of imaging in hypertrophic cardiomyopathy. It offers essential information regarding the presence, severity and location of LVH, the abnormalities of the mitral valve and subvalvular apparatus, left atrial enlargement, the systolic and diastolic function of the left ventricle and also evaluates the presence of left ventricular outflow tract obstruction [16,17].

### 2.1. Left Ventricular Wall Thickness

Left ventricular wall thickness should be measured at end diastole, perpendicular to the left ventricular cavity, in order to avoid the inclusion of left or right ventricular trabeculations [18,19]. The measurements are more accurate using bidimensional echo, in short-axis view at the apical level, papillary muscles and mitral valve [15]. The most common segment of hypertrophy is the basal interventricular septum, but considering the fact that often there are non-contiguous patterns of myocardial hypertrophy, all left ventricular segments should be evaluated from base to apex [20,21] The interventricular septum may have different morphologies: sigmoid, reverse curve, apical or neutral, morphologies, that will guide the type of septal reduction therapy. The presence of left ventricular hypertrophy, and especially an increased wall thickness (massive hypertrophy of ≥30 mm), is associated with a high risk of SCD [22]. The right ventricular wall thickness should also be measured, at end diastole, in subcostal or parasternal long-axis views, at the level of the tricuspid chordae, and its normal value is less than 5 mm [23].

When the 2-dimensional echo images are suboptimal and the severity of ventricular hypertrophy or the septal morphology is unclear, we may use the contrast imaging [24,25]. Contrast echocardiographic agents may also be used to assess the apical aneurysms in patients with apical HCM. In some cases, it is necessary to use the strain assessment, in order to see the myocardial mechanics and also to distinguish HCM from amyloidosis, considering the fact that in cardiac amyloidosis the apical strain is preserved, and the basal strain is significantly affected [18].

### 2.2. Mitral Valve and Subvalvular Apparatus Morphology and Mitral Hemodynamics

Mitral valve abnormalities are relatively common in patients with HCM, including an increased length of the mitral valve leaflets, sometimes even an important elongation of either posterior (≥17 mm in length) or anterior (≥30 mm in length) leaflet [26]. The increased length of the mitral valve leaflets may be responsible for the LVOT obstruction when one of them extends across from the coaptation point, in systole moving into the LVOT, to contact the interventricular septum [27]. The duration of mitral valve-interventricular septum contact during systole will influence the severity of LVOT gradient.

Subvalvular apparatus abnormalities, such as a hypertrophied anterolateral papillary muscle that is inserted directly on the anterior mitral leaflet, without chordae tendinea, can also cause a mid-cavity muscular obstruction, independent of systolic anterior motion (SAM). The presence of accessory papillary muscles or left ventricular muscle bundles can also cause outflow obstruction, by misplacing the mitral valve coaptation plane anteriorly, towards the interventricular septum [27,28]. As a secondary consequence of SAM, patients will have a mitral regurgitation, which is sometimes difficult to differentiate from LV outflow jet. Therefore, it is very important to analyze the Doppler systolic flow pattern, since in mitral regurgitation it begins abruptly in systole with increased velocities that persist in all systole, whereas the velocity from LVOT increases progressively in midsystole. The gravity of mitral regurgitation varies with the degree of LVOT obstruction and is typically inferolateral oriented [28,29,30]. If the mitral regurgitation jet is centrally directed, it is usually secondary to an endogenous mitral valve abnormality, so it may be necessary to use the transesophageal echocardiography for differential diagnosis [14,31].

### 2.3. Left Ventricular Outflow Obstruction

**Figure 1:**
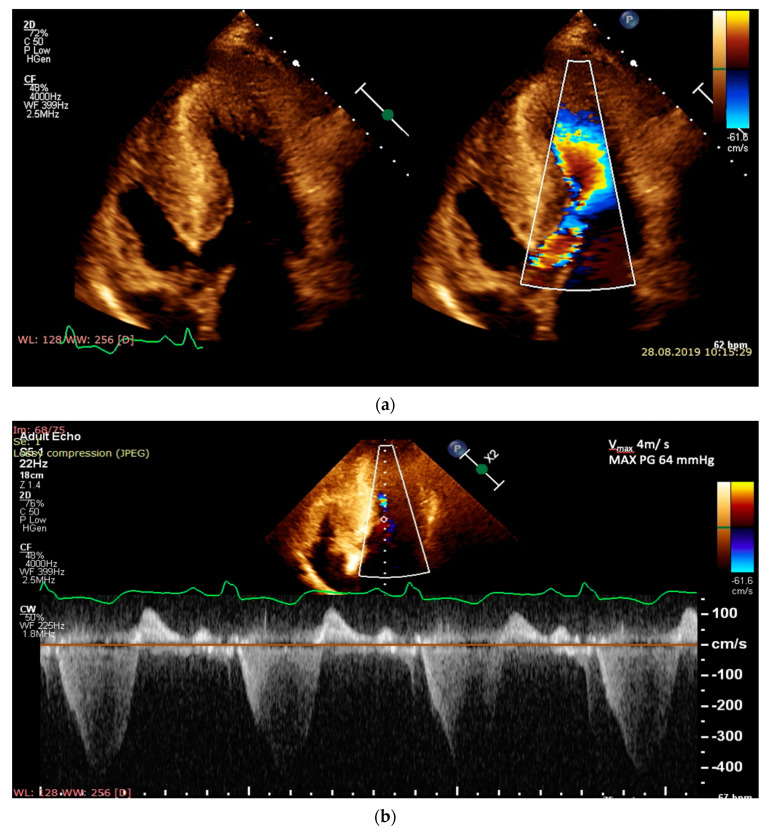
Echocardiography assessment at rest, in a 20-year-old male with obstructive hypertrophic cardiomyopathy: (**a**) apical five-chamber view with and without color Doppler demonstrating left ventricular mid-cavity and outflow tract obstruction, with turbulent flow; (**b**) continuous wave Doppler through the left ventricular outflow tract demonstrating the obstruction, with a peak gradient of 64 mmHg.

Left ventricular outflow obstruction appears secondary to the action of several complex mechanisms, represented by the reduced area of the left ventricular outflow tract, elongation of the anterior and/or posterior mitral leaflet, with an extension of them across from the coaptation point toward the interventricular septum and also the basal anteroseptal hypertrophy which bulges into LVOT [4,5,26]. Although the definition of HCM is based on the presence of LV hypertrophy, the LVOT obstruction causes heart failure symptoms and thus has become a defining element of HCM [14,32,33]. Dynamic LVOT obstruction may also appear in patients with hypertension, hypovolemia, calcification of the posterior mitral annulus or hypercontractile states. LVOT obstruction is defined as a doppler outflow tract pressure gradient ≥30 mmHg at rest or during provocation (Valsalva maneuver, standing or exercise) [2]. There are some data that sustain that post-prandial LVOT gradients are higher than those from the fasting state [34]. Assessment of LVOT obstruction is very important, not only for the management of symptoms but also to prevent sudden cardiac death [2]. The preferred method to induce LVOT obstruction, for symptomatic patients without resting LVOT obstruction, is exercise (stress) echocardiography. As only 50% of patients with provocable LVOT obstruction at stress echocardiography also have a positive response to the Valsalva maneuver, a normal response will not exclude the LVOT obstruction [31,33]. It is also recommended to avoid dobutamine for stress testing, because it may determine LVOT obstruction in healthy patients. A gradient in LVOT ≥50 mmHg is usually considered the limit at which LVOT obstruction is hemodynamically important and is necessary to initiate a treatment for septal reduction [14,23] (Figure 1).

It is known and already demonstrated by several studies, the association between the presence of LVOT obstruction, especially at rest, and the increased risk of sudden cardiac death [35,36]. In a recent study, it was highlighted that patients without resting LVOT obstruction, but with an increased provoked LVOT gradient of ≥90 mmHg, have worse outcomes compared to those with a low LVOT gradient <89 mmHg [37]. According to the protocol for the identification and treatment of LVOT obstruction, mentioned in the European Society of Cardiology Guideline [2], 2D and Doppler echocardiography at rest, during a Valsalva maneuver in the sitting or standing position (if no gradient is induced), is recommended in all patients with HCM [33,38]. If the maximum provoked LVOT gradient is <50 mmHg and the patient is asymptomatic, it is recommended to repeat echocardiography at 1 year. Exercise stress echocardiography for these patients should be considered when the existence of LVOT gradient is significant for lifestyle counseling or for choosing the optimal medical treatment. For patients with a maximum provoked peak LVOT gradient ≥50 mmHg, it is the recommended treatment of LVOT obstruction. This is also recommended for patients with maximum provoked LVOT gradient <50 mmHg who are symptomatic and, on exercise stress echocardiography, maximum provoked peak LVOT gradient increases ≥50 mmHg [2].

### 2.4. Left Atrial Enlargement

Most patients with HCM have a left atrial enlargement and its dimensions provide important information regarding the prognostic [39,40,41]. There are only a few studies that have evaluated the association between left atrial volume and the risk of SCD [42,43]. However, there are still debates about whether left atrial enlargement is an independent predictor of SCD, or if it appears as a result of LVH, LVOT obstruction, mitral regurgitation, diastolic dysfunction or atrial arrhythmia [44]. The most frequent causes for left atrial enlargement are the mitral regurgitation, as a secondary consequence of SAM, and increased left ventricular filling pressures [2].

### 2.5. Left Ventricular Ejection Fraction

It is recommended to assess the left ventricular ejection fraction (LVEF) using biplane method of discs, and in cases when we have a difficult imaging window or when it is difficult to visualize the endocardial border, we may use cardiac magnetic resonance (CMR) [44].

Approximately 10% of patients with nonobstructive HCM progress slowly to end-stage heart failure, being candidates for heart transplantation [44]. In addition, one half of them may progress to left ventricular enlargement and remodeling, with regression of ventricular hypertrophy due to the presence of diffuse myocardial fibrosis [45,46].

Patients with a left ventricular ejection fraction less than 50% often have a rapid clinical deterioration due to the microvascular dysfunction, which leads to a diffuse myocardial ischemia and fibrosis. Thus, for patients with HCM and a decreased left ventricular ejection fraction (≤50%), American College of Cardiology/ American Heart Association (ACC/AHA) guidelines highlight that implantable cardioverter defibrillator (ICD) implantation may be performed, considering their increased risk of adverse cardiac events [23]. Instead, the European Society of Cardiology did not include this statement in the current guidelines [14].

### 2.6. Diastolic Function

The diastolic dysfunction is highly prevalent in HCM patients. Almost one half of patients with HCM and preserved ejection fraction have symptoms of end-stage heart failure caused by diastolic dysfunction [45]. However, because echocardiography parameters used for the evaluation of left ventricular diastolic function, respectively, mitral inflow (E/A ratio) or tissue Doppler imaging-at the side of the mitral annulus, do not correlate with the results obtained from cardiac catheterization, they are not recommended as independent predictors of risk of end-stage heart failure or exercise duration [47]. Patients with HCM and a restrictive pattern of diastolic dysfunction, with E deceleration time <150 ms and E/A >2, have a poor prognosis [48]. One of the most important echocardiographic parameters that correlate best with left ventricular filling pressure and is used to assess diastolic function is the mitral early diastolic inflow to early diastolic tissue velocity (E/e’). It has recently been indicated that E/e’ may be used for risk stratification in HCM patients, a higher value of E/e’ being associated with worse event-free survival in nonobstructive HCM and in those with residual obstruction after myectomy [49].

### 2.7. Apical Aneurysm

Left ventricular apical aneurysm is defined as thin-walled, scared akinetic or dyskinetic wall segments, associated with both, midventricular and apical hypertrophy [50]. Apical hypertrophy is defined as the apical wall thickness ≥15 mm measured in and diastole, or maximal apical to basal wall thickness ratio ≥1.3 [51]. Left ventricular apical aneurisms may be small (transversal dimension between 1 to 2 cm) or large (>6 cm) and very rarely change in size over time. Apical aneurysm may result secondary to an increased apical pressure, myocardial wall stress and ischemia. Sometimes, there may appear ventricular tachyarrhythmias generated by the extension of the transmural scarring of the aneurysm into distal interventricular septum and/or left ventricular free wall [52]. The prevalence of apical aneurysm ranges between 1.3% and 4.8% [25,53], and, in most cases, its presence may be observed with echocardiography. However, in approximately 40% of cases, the presence of an apical aneurysm may be missed by echocardiography alone [50,54]. In these situations, we may use the contrast enhancement, which will define better the endocardium, the presence of an apical thrombus, myocardial fibrosis and scar [55]. Recent studies highlighted that patients with apical aneurysm have an increased risk of serious adverse events [25,50,56] and, thus, the 2011 ACC/AHA guidelines added apical aneurysm as a risk modifier for patients with HCM [23]. They have a high risk of sudden cardiac death and need to be considered for primary prevention implantable cardioverter-defibrillator and radiofrequency ablation for patients with recurrent ventricular tachyarrhythmias [52]. Additionally, these patients have an increased risk of thromboembolic stroke [25,50], but there are still not enough data to recommend initiating anticoagulant therapy in patients with apical aneurysm without an apical thrombus [14,23].

Echocardiography is the cornerstone investigation used for screening. During puberty, it is recommended to be carried out every 12 to 18 months and thereafter, to at least midlife, at every 3 to 5 years [4,5].

## 3. Myocardial Strain

**Figure 2:**
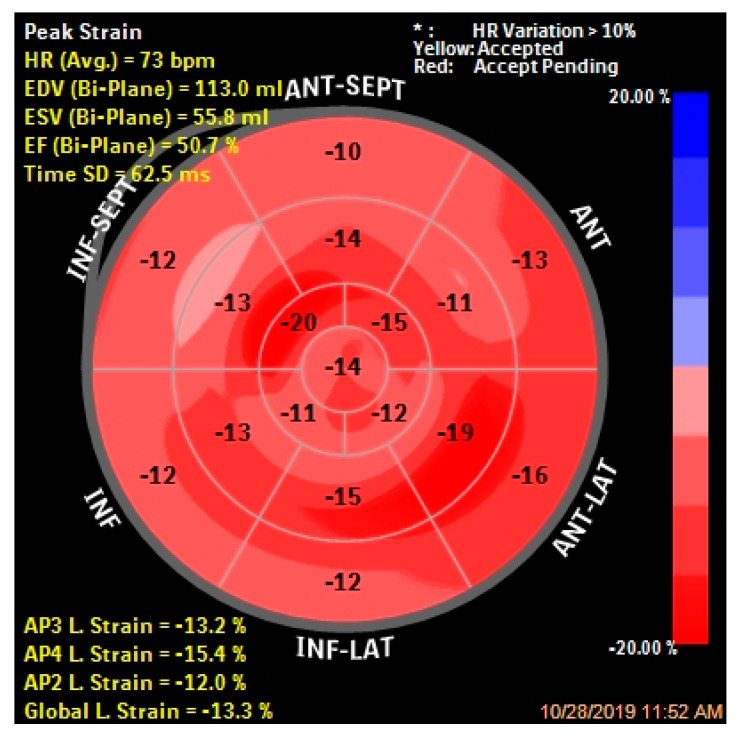
Longitudinal strain bull′s-eye plot in the same 20-year-old patient with obstructive hypertrophic cardiomyopathy. This patient has a reduced global longitudinal strain (GLS = −13.3%), in the presence of a normal left ventricular ejection fraction.

In patients with left ventricular hypertrophy, the left ventricular ejection fraction is not a suitable parameter for the evaluation of systolic function, considering that in cases of HCM it may be increased as a result of increase in radial thickening. Additionally, in the presence of left ventricular hypertrophy, the left ventricular cavity will be small which will cause a reduction in the stroke volume. In this context, a new and extremely important method to assess myocardial deformation and subsequently the left ventricular systolic function in patients with hypertrophic cardiomyopathy, is the two-dimensional strain. It derives from speckle tracking echocardiography and is a noninvasive and sensitive method that has been proposed as an additional test to differentiate physiological from pathological left ventricular hypertrophy [57,58]. There are three components of myocardial deformation—longitudinal, radial and circumferential. Global longitudinal strain (GLS) is an important parameter for clinical practice, being used for patients with preserved ejection fraction, in order to indicate the left ventricular subclinical dysfunction [59]. In patients with left ventricular hypertrophy, myocardial fiber disorganization and fibrosis affect the longitudinal function. Thus, in the early stages of the HCM, patients may have a reduced GLS, in the presence of a normal left ventricular ejection fraction (Figure 2). Additionally, relatives of HCM patients may have an impaired global longitudinal strain before the appearance of left ventricular hypertrophy [60,61]. In recent years, GLS has emerged as an important parameter for assessing the left ventricular function and some studies suggested its role as a clinical predictor of adverse cardiovascular events [62,63]. Thus, in a large prospective study the authors demonstrated that a reduced GLS value (GLS > −16%) was independently associated with a high risk of ventricular tachycardia/ventricular fibrillation, heart failure, cardiac transplantation and all-cause death. Further, GLS > −10% had significantly higher event rates, four times higher risk of events compared with GLS ≤ −16% (*p* = 0.006) [64].

This association between abnormal global longitudinal strain and adverse cardiac outcomes and ventricular arrhythmia was also demonstrated in a recent systematic review that included more than 3000 patients with HCM from a total of 14 observational studies. They observed that mean GLS was reduced, from −10% to −16%, in HCM patients and the lower the GLS values (less negative), the higher the risk of adverse cardiovascular events [65].

In another study, the authors evaluated the effects of myocardial hypertrophy and cardiac afterload on the myocardial deformation, at rest and during standardized exercise, for asymptomatic patients with moderate to severe aortic stenosis and patients with hypertrophic cardiomyopathy. They demonstrated that during submaximal exercise, longitudinal and circumferential LV deformations were lower in patients with aortic stenosis compared with those with HCM. These differences were not present at rest, and they can be explained by the greater afterload noticed in these patients, which reduces the contractile reserve. Thus, the authors highlighted that moderate exercise echocardiography shows impaired myocardial contractility, which is not obvious at rest, for patients with similar forms of left ventricular hypertrophy, respectively, patients with hypertrophic cardiomyopathy and aortic stenosis [66].

There are only a few studies that evaluated left ventricular segmental strain in patients with HCM and they demonstrated that regional LVH and myocardial fibrosis are independently and significantly associated with worse strain values [59,67]. However, considering the absence of prospective clinical studies; GLS cannot be used as a powerful clinical predictor of adverse cardiovascular events [65]. Further, there are some issues regarding the possibility of GLS to identify asymptomatic obstructive HCM patients or nonobstructive HCM patients at risk of end-stage heart failure, in order to initiate appropriate therapeutic strategies as early as possible.

## 4. Exercise Testing

Exercise testing is an essential noninvasive method for evaluation and for guiding treatment strategy in patients with HCM. There are various exercise testing methods that were previously underutilized in patients with HCM, and which have now become increasingly important.

Exercise (stress) echocardiography is more commonly used to assess left ventricular kinetic disorders in patients with suspicion of atherosclerotic coronary artery disease. Recently, this method has emerged as an important test for patients with HCM [68,69]. It predicts heart failure progression and also directs treatment strategies in patients with HCM. A recent study demonstrated that patients with no or mild baseline symptoms but with provocable obstruction during exercise testing, develop severe heart failure symptoms more frequently than those with nonobstructive HCM (3.2% per year vs. 1.6% per year, *p* = 0.002). In addition, patients with New York Heart Association functional class III/IV symptoms and provocable obstruction with exercise, are candidates for myectomy or alcohol septal ablation and those without obstruction are heart transplant candidates [70]. Thus, stress echocardiography predicts heart failure progression and also can make the differential diagnosis between obstructive and nonobstructive HCM, dictating different management strategies such as heart transplant for patients without obstruction, respectively, myectomy or alcohol septal ablation for those with gradients [68].

In order to measure oxygen capacity and thus evaluate the functional capacity, the cardiopulmonary (metabolic) exercise testing is very useful. The peak myocardial oxygen consumption with maximal exercise (Vo_2_) is used to identify high-risk patients, potential heart transplant candidates [71,72,73,74,75]. Thus, exercise testing has an important role in clarifying the long-term prognosis and choosing the optimal treatment for patients with HCM [68].

## 5. Computed Tomography Angiography

At least 25% of patients with hypertrophic cardiomyopathy may have chest pain, either on exertion or at rest [14,23,76]. There are many causes of angina in patients with HCM: microvascular ischemia caused by abnormal intramural coronary arterioles, loss of normal vasodilation reaction, which leads to a significant reduction in the coronary flow reserve, an increased oxygen demand on the hypertrophied myocardium and impaired left ventricular relaxation, which affects the coronary blood flow [14,23,76,77]. As only 50% of patients with HCM and coronary artery disease may have evident left ventricular kinetic disorders at exercise echocardiography, computed tomography angiography represents a noninvasive alternative for patients with lower risk [23]. For symptomatic patients with moderate-to-high risk of coronary artery disease, coronary angiography is still the recommended test [77]. It has recently been indicated that preprocedural computed tomography imaging has a significant impact on localization of the appropriate target septal artery for alcohol septal ablation in HCM patients and to limit the area of myocardial necrosis [78].

## 6. Cardiac Nuclear Imaging

Although it is not an investigation frequently used in clinical practice for patients with hypertrophic cardiomyopathy, positron emission tomography allows evaluation of myocardial blood flow. In patients without epicardial coronary artery disease, positron emission tomography may be used as a marker for microvascular dysfunction. Studies highlighted that patients with the most significant impairment of the myocardial blood flow have an increased risk of end-stage heart failure and cardiovascular mortality [79].

## 7. Cardiac Magnetic Resonance

Cardiac magnetic resonance has an important role in diagnosis, risk stratification and management strategies of HCM patients, considering its high spatial resolution, better contrast between blood and myocardium, accurate volumetric evaluation of cardiac chambers and also the fact that it is not influenced by the chest anatomy and associated pulmonary parenchymal pathologies, compared to echocardiography. It is considered the gold standard for the evaluation of wall thickness and chamber volumes, allowing a more accurate characterization of heart volumes and functions, tissue morphology and extension of myocardial hypertrophy [80,81]. It is an excellent imaging technique to detect apical aneurysms, clots or papillary muscle. According to the European Society of Cardiology guidelines [15], CMR should be performed, at least, as an initial evaluation, for all HCM patients, if local resources and expertise permit. Cardiac magnetic resonance also identifies the extension of left ventricular hypertrophy, which may be focal (1–2 hypertrophic segments, the least common), intermediate (3–7 hypertrophic segments) and diffuse (8–16 hypertrophic segment, the most common) [2].

CMR sequences used for the evaluation of myocardial pathologies can be divided into tissue characterization sequences and functional assessment. Thus, late gadolinium enhancement (LGE) method can detect the presence and type of LGE: Cine sequences are used for the evaluation of systolic function (ejection fraction), ventricular volumes and wall morphology; T2* imaging detects the iron deposition; T1 mapping is used for diffuse myocardial fibrosis and T2 mapping for tissue edema [2,80].

**Figure 3:**
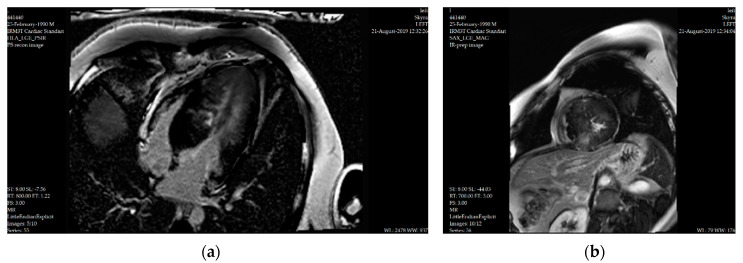
Late gadolinium enhancement images: (**a**) four-chamber view and (**b**) short-axis view, with diffuse nonischemic fibrotic lesions in the left ventricular myocardium and intramural nodular fibrotic lesions in the interventricular septum, in the same 20-year-old patient.

Of all the CMR sequences, LGE images have the most important role in tissue characterization in HCM patients. By using gadolinium-based contrast agents, late gadolinium enhancement method ensures a high spatial resolution (less or equal than 1 mm in plane). It can detect with great accuracy small areas of myocardial fibrosis, myocardial disarray and scarring. LGE is found in about two-thirds of HCM patients, frequently in areas of hypertrophy or right ventricular insertion points [81] (Figure 3).

In recent years, there has been increased recognition that the presence and extent of LGE on CMR is an important risk factor for predicting sudden cardiac death in HCM patients. Multiple studies and meta-analyses demonstrated a high risk of cardiovascular mortality, heart failure and all-cause death in the presence of LGE in HCM patients [14,65,82,83].

In a meta-analysis performed by Green et al., they included almost 1100 HCM patients from four studies, over an average follow-up of 3.1 years. They attempted to evaluate the risk of adverse events associated with the presence of LGE. Their results highlighted that the presence of LGE was statistically significantly associated with cardiac death, heart failure death and all-cause mortality in HCM patients, but they had no data regarding the extent of LGE [83].

Another important meta-analysis that evaluated the predictive value of LGE-CMR for adverse events and death in HCM patients was published by Weng and coworkers. The meta-analysis included almost 3000 patients over a median follow-up of 3.1 years and the authors demonstrated that the extent of LGE is significantly associated with a high risk of SCD, even after adjusting for baseline characteristics (Hazard Ratio (HR) adjusted: 1.36/10% LGE; 95% CI: 1.10 to 1.69; *p* = 0.005). The presence of LGE was also associated with a high risk of all-cause mortality, cardiovascular mortality and a trend for heart failure death [65].

In another recent study, Mentias et al. aimed to assess the prognostic utility of LGE in patients with HCM with low/intermediate sudden cardiac death risk and preserved left ventricular ejection fraction. They demonstrated that in patients with HCM (obstructive, myectomy and nonobstructive) with preserved LVEF and low/intermediate risk, the extent of LGE was statistically significantly associated with a higher rate of composite endpoints, giving incremental prognostic utility [84].

The role of LGE-CMR in improving risk stratification strategies in HCM patients was also studied by Freitas et al. During a median follow-up of 3.4 years, their results suggest that the amount of LGE has an important prognostic value and that using LGE for sudden cardiac death risk stratification would correctly reclassify a considerable number of patients [80].

All these studies demonstrated that LGE is an independent predictor of sudden cardiac death in HCM patients and may be considered useful in identifying patients who will most benefit from implantation of a cardioverter-defibrillator (ICD) for the primary prevention of sudden cardiac death [14,65,82,83,84].

T1 mapping is a novel CMR technique used to measure the extracellular volume fraction and for further identification of diffuse interstitial fibrosis. Even though there are limited data regarding the association between increased native T1 times and extracellular volume with adverse outcomes, more studies suggest its clinical utility for differentiating HCM from Fabry disease, cardiac amyloidosis or hypertensive heart disease [85,86,87].

T2* mapping is able to detect ischemic segments, and in HCM patients potentially triggered through relative ischemia, they have had reduced T2* values [88]. In a recent retrospective study, the authors aimed to evaluate the risk of arrhythmia and heart failure in HCM patients, in relation with myocardial T2* mapping. Especially in non-obstructive HCM patients, it was observed that low T2* values are minimally associated with arrhythmic events. There was no association between T2* with heart failure, thus myocardial fibrosis by LGE remains the strongest predictor, and T2* mapping may be used only in certain clinical contexts [89].

In Table 1, we summarized the indications and therapeutic implications of multimodality imaging in choosing the optimal treatment for patients with hypertrophic cardiomyopathy. There are also other new techniques, such as CMR diffusion tensor imaging, but they are not yet ready for use in clinical practice.

## 8. Conclusions

Multimodality imaging is necessary to assess the complete morphology of hypertrophic cardiomyopathy and has an important role in choosing the optimal treatment and in the long-term prognosis of patients with HCM. Considering that patients with HCM that are at low risk, according to the current risk stratification models, may have adverse events, it is necessary to identify additional markers that can be used for predicting adverse events and, thus, choosing the optimal treatment. We believe that all the recent developments in the multimodality imaging for patients with HCM, will significantly transform their treatment in the coming years, considering the recent developments in gene-based therapies and minimally invasive procedural techniques. The use of new multimodality imaging methods, such as cardiac magnetic resonance, myocardial strain, computed tomography angiography or positron emission tomography, allows an improvement of the therapeutic strategies of HCM patients, which will improve their prognosis and quality of life.

## Figures and Tables

**Table 1:** Indications and therapeutic implications of multimodality imaging in choosing the optimal treatment in hypertrophic cardiomyopathy.

Multimodality Imaging	Indications	Therapeutic Implications
**Echocardiography**	- first line technique in all HCM patients; - should be performed every 1–2 years, in clinically stable patients; - evaluates the ***left ventricle*** (wall thickness, cavity size, systolic and diastolic function, left ventricular outflow tract obstruction), ***mitral valve*** (systolic anterior motion of the mitral valve, leaflets, chordae and papillary muscles abnormalities, severity of mitral regurgitation) and ***aortic valve*** (leaflet sclerosis, mid-systolic partial valve closure).	- patients with massive hypertrophy (wall thickness ≥ 30 mm) have an increased risk of arrhythmic SCD and primary prevention ICDs are effective. They may also develop progressive drug-refractory HF, but this is reversible in most cases, after surgical myectomy. - GLS identifies patients with subclinical left ventricular dysfunction in the presence of preserved ejection fraction.
**Cardiac magnetic resonance**	- should be performed at the initial evaluation, if it is possible; - used for LGE/fibrosis assessment; anatomical assessment before invasive gradient reduction therapy; differential diagnosis or in case of suboptimal echo images.	- extensive LGE identifies patients at risk of SCD (they deserve consideration for primary prevention ICDs) and patients at risk of end-stage HF (require close clinical surveillance); - in 40% of cases, the apical aneurysm may be missed by echocardiography and we may use contrast enhancement. These patients have an increased risk of serious adverse events and need to be considered for primary prevention ICDs and radiofrequency ablation for those with recurrent ventricular tachyarrhythmia.
**Computed tomography angiography**	- used for anatomical assessment of the epicardial coronary arteries (bridging, epicardial CAD) or in case of suboptimal echo images and contraindications for cardiac magnetic resonance.	- is important in localization of the appropriate target septal artery for alcohol septal ablation and to limit the area of myocardial necrosis.
**Cardiac nuclear imaging**	- to assess the myocardial perfusion; metabolism, receptors and innervation; differential diagnosis or in case of suboptimal echo images and contraindications for cardiac magnetic resonance.	- patients with the most significant impairment of the myocardial blood flow have an increased risk of end-stage HF and cardiovascular mortality.
**Cardiopulmonary exercise testing**	- used to assess functional capacity by evaluating O_2_ capacity.	- is relevant for nonobstructive HCM patients with end-stage HF that are candidates for a heart transplant and for patients with obstructive HCM that require invasive septal reduction therapy.

CAD, coronary artery disease; GLS, global longitudinal strain; HCM, hypertrophic cardiomyopathy; HF, heart failure; ICDs, implantable cardioverter-defibrillator; LGE, late gadolinium enhancement; SCD, sudden cardiac death.

## References

[1] Tuohy C.V., Kaul S., Song H.K., Nazer B., Heitner S.B. (2020). Hypertrophic cardiomyopathy: The future of treatment. Eur. J. Heart Fail..

[2] Elliott P.M., Anastasakis A., Borger M.A., Borggrefe M., Cecchi F., Charron P., Hagege A.A., Lafont A., Limongelli G., Mahrholdt H. (2014). 2014 ESC guidelines on diagnosis and management of hypertrophic cardiomyopathy: The Task Force for the diagnosis and management of hypertrophic cardiomyopathy of the European Society of Cardiology (ESC). Eur. Heart J..

[3] Makavos G., Κairis C., Tselegkidi M.-E., Karamitsos T., Rigopoulos A.G., Noutsias M., Ikonomidis I. (2019). Hypertrophic cardiomyopathy: An updated review on diagnosis, prognosis, and treatment. Heart Fail. Rev..

[4] Maron B.J. (2018). Clinical course and management of hypertrophic cardiomyopathy. N. Engl. J. Med..

[5] Spudich J.A. (2019). Three perspectives on the molecular basis of hypercontractility caused by hypertrophic cardiomyopathy mutations. Pflug. Arch..

[6] Maron B.J., Gardin J.M., Flack J.M., Gidding S.S., Kurosaki T.T., Bild D.E. (1995). Prevalence of hypertrophic cardiomyopathy in a general population of young adults: Echocardiographic analysis of 4111 subjects in the CARDIA study. Circulation.

[7] Ramchand J., Fava A.M., Chetrit M., Desai M.Y. (2020). Advanced imaging for risk stratification of sudden death in hypertrophic cardiomyopathy. Heart.

[8] Rowin E.J., Maron B.J., Maron M.S. (2019). The hypertrophic cardiomyopathy phenotype viewed through the prism of multimodality imaging: Clinical and etiologic implications. JACC Cardiovasc. Imaging.

[9] Zou Y., Song L., Wang Z., Ma A., Liu T., Gu H., Lu S., Wu P., Zhang Y., Shen L. (2004). Prevalence of idiopathic hypertrophic cardiomyopathy in China: A population-based echocardiographic analysis of 8080 adults. Am. J. Med..

[10] Teare D. (1958). Asymmetrical hypertrophy of the heart in young adults. Br. Heart J..

[11] Braunwald E., Lambrew C.T., Rockoff S.D., Ross J., Morrow A.G. (1964). Idiopathic hypertrophic subaortic stenosis: A description of the disease based upon an analysis of 64 patients. Circulation.

[12] Teekakirikul P., Zhu W., Huang H.C., Fung E. (2019). Hypertrophic cardiomyopathy: An overview of genetics and management. Biomolecules.

[13] Weissler-Snir A., Allan K., Cunningham K., Connelly K.A., Lee D.S., Spears D.A., Rakowski H., Dorian P. (2019). Hypertrophic cardiomyopathy–related sudden cardiac death in young people in Ontario. Circulation.

[14] Marian A.J., Braunwald E. (2017). Hypertrophic cardiomyopathy: Genetics, pathogenesis, clinical manifestations, diagnosis, and therapy. Circ. Res..

[15] Cardim N., Galderisi M., Edvardsen T., Plein S., Popescu B.A., d’Andrea A., Bruder O., Cosyns B., Davin L., Donal E. (2015). Role of multimodality cardiac imaging in the management of patients with hypertrophic cardiomyopathy: An expert consensus of the European Association of Cardiovascular Imaging Endorsed by the Saudi Heart Association. Eur. Heart J. Cardiovasc. Imag..

[16] Geske J.B., Ommen S.R., Gersh B.J. (2018). Hypertrophic cardiomyopathy: Clinical update. JACC Heart Fail..

[17] Maron B.J., Rowin E.J., Casey S.A., Maron M.S. (2016). How hypertrophic cardiomyopathy became a contemporary treatable genetic disease with low mortality: Shaped by 50 years of clinical research and practice. JAMA Cardiol..

[18] Bois J.P., Geske J.B., Foley T.A., Ommen S.R., Pellikka P.A. (2017). Comparison of maximal wall thickness in hypertrophic cardiomyopathy differs between magnetic resonance imaging and transthoracic echocardiography. Am. J. Cardiol..

[19] Phelan D., Sperry B.W., Thavendiranathan P., Collier P., Popović Z.B., Lever H.M., Smedira N.G., Desai M.Y. (2017). Comparison of ventricular septal measurements in hypertrophic cardiomyopathy patients who underwent surgical myectomy using multimodality imaging and implications for diagnosis and management. Am. J. Cardiol..

[20] Maron M.S., Maron B.J., Harrigan C., Buros J., Gibson C.M., Olivotto I., Biller L., Lesser J.R., Udelson J.E., Manning W.J. (2009). Hypertrophic cardiomyopathy phenotype revisited after 50 years with cardiovascular magnetic resonance. J. Am. Coll. Cardiol..

[21] Maron B.J., Epstein S.E. (1986). Clinical significance and therapeutic implications of the left ventricular outflow tract pressure gradient in hypertrophic cardiomyopathy. Am. J. Cardiol..

[22] Maron M.S., Maron B.J. (2015). Clinical impact of contemporary cardiovascular magnetic resonance imaging in hypertrophic cardiomyopathy. Circulation.

[23] Gersh Bernard J., Maron Barry J., Bonow Robert O., Dearani Joseph A., Fifer Michael A., Link Mark S., Naidu Srihari S., Nishimura Rick A., Ommen Steve R., Rakowski Harry S.C.E. (2011). ACCF/AHA Guideline for the diagnosis and treatment of hypertrophic cardiomyopathy: A report of the American College of Cardiology Foundation/American Heart Association Task Force on Practice Guidelines. Circulation.

[24] Klarich K.W., Attenhofer Jost C.H., Binder J., Connolly H.M., Scott C.G., Freeman W.K., Ackerman M.J., Nishimura R.A., Tajik A.J., Ommen S.R. (2013). Risk of death in long-term follow-up of patients with apical hypertrophic cardiomyopathy. Am. J. Cardiol..

[25] Rowin E.J., Maron B.J., Haas T.S., Garberich R.F., Wang W., Link M.S., Maron M.S. (2017). Hypertrophic cardiomyopathy with left ventricular apical aneurysm: Implications for risk stratification and management. J. Am. Coll. Cardiol..

[26] Olivotto I., Hellawell J.L., Farzaneh-Far R., Blair C., Coppini R., Myers J., Belardinelli L., Maron M.S. (2016). Novel approach targeting the complex pathophysiology of hypertrophic cardiomyopathy: The impact of late sodium current inhibition on exercise capacity in subjects with symptomatic hypertrophic cardiomyopathy (LIBERTY-HCM) trial. Circ. Heart Fail..

[27] Nishimura R.A., Seggewiss H., Schaff H.V. (2017). Hypertrophic obstructive cardiomyopathy: Surgical myectomy and septal ablation. Circ. Res..

[28] Agarwal S., Tuzcu E.M., Desai M.Y., Smedira N., Lever H.M., Lytle B.W., Kapadia S.R. (2010). Updated meta-analysis of septal alcohol ablation versus myectomy for hypertrophic cardiomyopathy. J. Am. Coll. Cardiol..

[29] Wigle E.D. (2001). Cardiomyopathy: The diagnosis of hypertrophic cardiomyopathy. Heart.

[30] Sherrid M.V., Wever-Pinzon O., Shah A., Chaudhry F.A. (2009). Reflections of inflections in hypertrophic cardiomyopathy. J. Am. Coll. Cardiol..

[31] Veselka J., Anavekar N.S., Charron P. (2017). Hypertrophic obstructive cardiomyopathy. Lancet.

[32] Kramer C.M., Appelbaum E., Desai M.Y., Desvigne-Nickens P., DiMarco J.P., Friedrich M.G., Geller N., Heckler S., Ho C.Y., Jerosch-Herold M. (2015). Hypertrophic cardiomyopathy registry: The rationale and design of an international, observational study of hypertrophic cardiomyopathy. Am. Heart J..

[33] Maron M.S., Olivotto I., Zenovich A.G., Link M.S., Pandian N.G., Kuvin J.T., Nistri S., Cecchi F., Udelson J.E., Maron B.J. (2006). Hypertrophic cardiomyopathy is predominantly a disease of left ventricular outflow tract obstructio. Circulation.

[34] Nistri S., Olivotto I., Maron M.S., Ferrantini C., Coppini R., Grifoni C., Baldini K., Sgalambro A., Cecchi F., Maron B.J. (2012). β Blockers for prevention of exercise-induced left ventricular outflow tract obstruction in patients with hypertrophic cardiomyopathy. Am. J. Cardiol..

[35] Maron M.S., Olivotto I., Betocchi S., Casey S.A., Lesser J.R., Losi M.A., Cecchi F., Maron B.J. (2003). Effect of left ventricular outflow tract obstruction on clinical outcome in hypertrophic cardiomyopathy. N. Engl. J. Med..

[36] Gimeno J.R., Tomé-Esteban M., Lofiego C., Hurtado J., Pantazis A., Mist B., Lambiase P., McKenna W.J., Elliott P.M. (2009). Exercise-induced ventricular arrhythmias and risk of sudden cardiac death in patients with hypertrophic cardiomyopathy. Eur. Heart J..

[37] Lu D.-Y., Hailesealassie B., Ventoulis I., Liu H., Liang H.-Y., Nowbar A., Pozios I., Canepa M., Cresswell K., Luo H.C. (2017). Impact of peak provoked left ventricular outflow tract gradients on clinical outcomes in hypertrophic cardiomyopathy. Int. J. Cardiol..

[38] Dimitrow P.P., Bober M., Michałowska J., Sorysz D. (2009). Left ventricular outflow tract gradient provoked by upright position or exercise in treated patients with hypertrophic cardiomyopathy without obstruction at rest. Echocardiography.

[39] Guttmann O.P., Rahman M.S., O’Mahony C., Anastasakis A., Elliott P.M. (2014). Atrial fibrillation and thromboembolism in patients with hypertrophic cardiomyopathy: Systematic review. Heart.

[40] O’Mahony C., Jichi F., Pavlou M., Monserrat L., Anastasakis A., Rapezzi C., Biagini E., Gimeno J.R., Limongelli G., McKenna W.J. (2014). A novel clinical risk prediction model for sudden cardiac death in hypertrophic cardiomyopathy (HCM risk-SCD). Eur. Heart J..

[41] Spirito P., Autore C., Rapezzi C., Bernabò P., Badagliacca R., Maron M.S., Bongioanni S., Coccolo F., Estes N.A.M., Barillà C.S. (2009). Syncope and risk of sudden death in hypertrophic cardiomyopathy. Circulation.

[42] Yang W.I., Shim C.Y., Kim Y.J., Kim S.A., Rhee S.J., Choi E.Y., Choi D., Jang Y., Chung N., Cho S.Y. (2009). Left atrial volume index: A predictor of adverse outcome in patients with hypertrophic cardiomyopathy. J. Am. Soc. Echocardiogr..

[43] Debonnaire P., Thijssen J., Leong D.P., Joyce E., Katsanos S., Hoogslag G.E., Schalij M.J., Atsma D.E., Bax J.J., Delgado V. (2014). Global longitudinal strain and left atrial volume index improve prediction of appropriate implantable cardioverter defibrillator therapy in hypertrophic cardiomyopathy patients. Int. J. Cardiovasc. Imaging.

[44] Tower-Rader A., Kramer C.M., Neubauer S., Nagueh S.F., Desai M.Y. (2020). Multimodality imaging in hypertrophic cardiomyopathy for risk stratification. Circ. Cardiovasc. Imaging.

[45] Toepfer C.N., Wakimoto H., Garfinkel A.C., McDonough B., Liao D., Jiang J., Tai A.C., Gorham J.M., Lunde I.G., Lun M. (2019). Hypertrophic cardiomyopathy mutations in MYBPC3 dysregulate myosin. Sci. Transl. Med..

[46] Penicka M., Gregor P., Kerekes R., Marek D., Curila K., Krupicka J. (2009). Candesartan use in Hypertrophic And Non-obstructive Cardiomyopathy Estate (CHANCE) Study Investigators. The effects of candesartan on left ventricular hypertrophy and function in nonobstructive hypertrophic cardiomyopathy: A pilot, randomized study. J. Mol. Diagn..

[47] Araujo A.Q., Arteaga E., Ianni B.M., Buck P.C., Rabello R., Mady C. (2005). Effect of Losartan on left ventricular diastolic function in patients with nonobstructive hypertrophic cardiomyopathy. Am. J. Cardiol..

[48] Biagini E., Spirito P., Rocchi G., Ferlito M., Rosmini S., Lai F., Lorenzini M., Terzi F., Bacchi-Reggiani L., Boriani G. (2009). Prognostic implications of the Doppler restrictive filling pattern in hypertrophic cardiomyopathy. Am. J. Cardiol..

[49] Lu D.-Y., Haileselassie B., Ventoulis I., Liu H.-Y., Liang H.-Y., Pozios I., Canepa M., Phillip S., Abraham M.R., Abraham T. (2018). E/e′ ratio and outcome prediction in hypertrophic cardiomyopathy: The influence of outflow tract obstruction. Eur. Heart J. Cardiovasc. Imaging.

[50] Maron M.S., Finley J.J., Bos J.M., Hauser T.H., Manning W.J., Haas T.S., Lesser J.R., Udelson J.E., Ackerman M.J., Maron B.J. (2008). Prevalence, clinical significance, and natural history of left ventricular apical aneurysms in hypertrophic cardiomyopathy. Circulation.

[51] Suzuki J., Shimamoto R., Nishikawa J., Yamazaki T., Tsuji T., Nakamura F., Shin W.S., Nakajima T., Toyo-Oka T., Ohotomo K. (1999). Morphological onset and early diagnosis in apical hypertrophic cardiomyopathy: A long-term analysis with nuclear magnetic resonance imaging. J. Am. Coll. Cardiol..

[52] Nijenkamp L.L.A.M., Bollen I.A.E., van Velzen H.G., Regan J.A., van Slegtenhorst M., Niessen H.W.M., Schinkel A.F.L., Krüger M., Poggesi C., Ho C.Y. (2018). Sex differences at the time of myectomy in hypertrophic cardiomyopathy. Circ. Heart Fail..

[53] Xiao Y., Wang L.P., Yang Y.K., Tian T., Yang K.Q., Sun X., Jiang Y., Liu Y.X., Zhou X.L., Li J. (2016). Clinical profile and prognosis of left ventricular apical aneurysm in hypertrophic cardiomyopathy. Am. J. Med. Sci..

[54] Lee S.P., Park K., Kim H.K., Kim Y.J., Sohn D.W. (2013). Apically displaced papillary muscles mimicking apical hypertrophic cardiomyopathy. Eur. Heart J. Cardiovasc. Imaging.

[55] To A.C.Y., Dhillon A., Desai M.Y. (2011). Cardiac magnetic resonance in hypertrophic cardiomyopathy. JACC Cardiovasc. Imaging.

[56] Ichida M., Nishimura Y., Kario K. (2014). Clinical significance of left ventricular apical aneurysms in hypertrophic cardiomyopathy patients: The role of diagnostic electrocardiography. J. Cardiol..

[57] Serri K., Reant P., Lafitte M., Berhouet M., Le Bouffos V., Roudaut R., Lafitte S. (2006). Global and regional myocardial function quantification by two-dimensional strain: Application in hypertrophic cardiomyopathy. J. Am. Coll. Cardiol..

[58] Kansal M.M., Lester S.J., Surapaneni P., Sengupta P.P., Appleton C.P., Ommen S.R., Ressler S.W., Hurst R.T. (2011). Usefulness of two-dimensional and speckle tracking echocardiography in “gray zone” left ventricular hypertrophy to differentiate professional football player’s heart from hypertrophic cardiomyopathy. Am. J. Cardiol..

[59] Collier P., Phelan D., Klein A. (2017). A test in context: Myocardial strain measured by speckle-tracking echocardiography. J. Am. Coll. Cardiol..

[60] Yang H., Carasso S., Woo A., Jamorski M., Nikonova A., Wigle E.D., Rakowski H. (2010). Hypertrophy pattern and regional myocardial mechanics are related in septal and apical hypertrophic cardiomyopathy. J. Am. Soc. Echocardiogr..

[61] Urbano-Moral J.A., Rowin E.J., Maron M.S., Crean A., Pandian N.G. (2014). Investigation of global and regional myocardial mechanics with 3-dimensional speckle tracking echocardiography and relations to hypertrophy and fibrosis in hypertrophic cardiomyopathy. Circ. Cardiovasc. Imaging.

[62] Paraskevaidis I.A., Farmakis D., Papadopoulos C., Ikonomidis I., Parissis J., Rigopoulos A., Iliodromitis E.K., Kremastinos D.T. (2009). Two-dimensional strain analysis in patients with hypertrophic cardiomyopathy and normal systolic function: A 12-month follow-up study. Am. Heart J..

[63] Tower-Rader A., Mohananey D., To A., Lever H.M., Popovic Z.B., Desai M.Y. (2019). Prognostic value of global longitudinal strain in hypertrophic cardiomyopathy: A systematic review of existing literature. JACC Cardiovasc. Imaging.

[64] Liu H., Pozios I., Haileselassie B., Nowbar A., Sorensen L.L., Phillip S., Lu D.-Y., Ventoulis I., Luo H., Abraham M.R. (2017). Role of global longitudinal strain in predicting outcomes in hypertrophic cardiomyopathy. Am. J. Cardiol..

[65] Weng Z., Yao J., Chan R.H., He J., Yang X., Zhou Y., He Y. (2016). Prognostic value of LGE-CMR in HCM: A meta-analysis. JACC Cardiovasc. Imaging.

[66] Schnell F., Donal E., Bernard-Brunet A., Reynaud A., Wilson M.G., Thebault C., Ridard C., Mabo P., Carré F. (2013). Strain analysis during exercise in patients with left ventricular hypertrophy: Impact of etiology. J. Am. Soc. Echocardiogr..

[67] Popović Z.B., Kwon D.H., Mishra M., Buakhamsri A., Greenberg N.L., Thamilarasan M., Flamm S.D., Thomas J.D., Lever H.M., Desai M.Y. (2008). Association between regional ventricular function and myocardial fibrosis in hypertrophic cardiomyopathy assessed by speckle tracking echocardiography and delayed hyperenhancement magnetic resonance imaging. J. Am. Soc. Echocardiogr..

[68] Rowin E.J., Maron B.J., Olivotto I., Maron M.S. (2017). Role of exercise testing in hypertrophic cardiomyopathy. JACC Cardiovasc. Imaging.

[69] Desai M.Y., Bhonsale A., Patel P., Naji P., Smedira N.G., Thamilarasan M., Lytle B.W., Lever H.M. (2014). Exercise echocardiography in asymptomatic HCM: Exercise capacity and not LV outflow tract gradient predicts long-term outcomes. JACC Cardiovasc. Imaging.

[70] Coats C.J., Rantell K., Bartnik A., Patel A., Mist B., McKenna W.J., Elliott P.M. (2015). Cardiopulmonary exercise testing and prognosis in hypertrophic cardiomyopathy. Circ. Heart Fail..

[71] Finocchiaro G., Haddad F., Knowles J.W., Caleshu C., Pavlovic A., Homburger J., Shmargad Y., Sinagra G., Magavern E., Wong M. (2015). Cardiopulmonary responses and prognosis in hypertrophic cardiomyopathy: A potential role for comprehensive noninvasive hemodynamic assessment. JACC Heart Fail..

[72] Sorajja P., Allison T., Hayes C., Nishimura R.A., Lam C.S.P., Ommen S.R. (2012). Prognostic utility of metabolic exercise testing in minimally symptomatic patients with obstructive hypertrophic cardiomyopathy. Am. J. Cardiol..

[73] Masri A., Pierson L.M., Smedira N.G., Agarwal S., Lytle B.W., Naji P., Thamilarasan M., Lever H.M., Cho L.S., Desai M.Y. (2015). Predictors of long-term outcomes in patients with hypertrophic cardiomyopathy undergoing cardiopulmonary stress testing and echocardiography. Am. Heart J..

[74] Magrì D., Re F., Limongelli G., Agostoni P., Zachara E., Correale M., Mastromarino V., Santolamazza C., Casenghi M., Pacileo G. (2016). Heart failure progression in hypertrophic cardiomyopathy—Possible insights from cardiopulmonary exercise testing. Circ. J..

[75] Magrì D., Limongelli G., Re F., Agostoni P., Zachara E., Correale M., Mastromarino V., Santolamazza C., Casenghi M., Pacileo G. (2016). Cardiopulmonary exercise test and sudden cardiac death risk in hypertrophic cardiomyopathy. Heart.

[76] Maron B.J., Ommen S.R., Semsarian C., Spirito P., Olivotto I., Maron M.S. (2014). Hypertrophic cardiomyopathy: Present and future, with translation into contemporary cardiovascular medicine. J. Am. Coll. Cardiol..

[77] Raphael C.E., Cooper R., Parker K.H., Collinson J., Vassiliou V., Pennell D.J., de Silva R., Hsu L.Y., Greve A.M., Nijjer S. (2016). Mechanisms of myocardial ischaemia in hypertrophic cardiomyopathy: Insights from wave intensity analysis and magnetic resonance. J. Am. Coll. Cardiol..

[78] Yanagiuchi T., Tada N., Haga Y., Suzuki S., Sakurai M., Taguri M., Ootomo T. (2019). Utility of preprocedural multidetector computed tomography in alcohol septal ablation for hypertrophic obstructive cardiomyopathy. Cardiovasc. Interv. Ther..

[79] Olivotto I., Cecchi F., Gistri R., Lorenzoni R., Chiriatti G., Girolami F., Torricelli F., Camici P.G. (2006). Relevance of coronary microvascular flow impairment to long-term remodelling and systolic dysfunction in hypertrophic cardiomyopathy. J. Am. Coll. Cardiol..

[80] Freitas P., Ferreira A.M., Arteaga-Fernández E., de Oliveira Antunes M., Mesquita J., Abecasis J., Marques H., Saraiva C., Matos D.N., Rodrigues R. (2019). The amount of late gadolinium enhancement outperforms current guideline-recommended criteria in the identification of patients with hypertrophic cardiomyopathy at risk of sudden cardiac death. J. Cardiovasc. Magn. R..

[81] Conte M.R., Bongioanni S., Chiribiri A., Leuzzi S., Lardone E., Di Donna P., Pizzuti A., Luceri S., Cesarani F., Mabritto B. (2011). Late gadolinium enhancement on cardiac magnetic resonance and phenotypic expression in hypertrophic cardiomyopathy. Am. Heart J..

[82] Bruder O., Wagner A., Jensen C.J., Schneider S., Ong P., Kispert E.M., Nassenstein K., Schlosser T., Sabin G.V., Sechtem U. (2010). Myocardial scar visualized by cardiovascular magnetic resonance imaging predicts major adverse events in patients with hypertrophic cardiomyopathy. J. Am. Coll. Cardiol..

[83] Green J.J., Berger J.S., Kramer C.M., Salerno M. (2012). Prognostic value of late gadolinium enhancement in clinical outcomes for hypertrophic cardiomyopathy. JACC Cardiovasc. Imaging.

[84] Mentias A., Raeisi-Giglou P., Smedira N.G., Feng K., Sato K., Wazni O., Kanj M., Flamm S.D., Thamilarasan M., Popovic Z.B. (2018). Late gadolinium enhancement in patients with hypertrophic cardiomyopathy and preserved systolic function. J. Am. Coll. Cardiol..

[85] Avanesov M., Münch J., Weinrich J., Well L., Säring D., Stehning C., Tahir E., Bohnen S., Radunski U.K., Muellerleile K. (2017). Prediction of the estimated 5-year risk of sudden cardiac death and syncope or non-sustained ventricular tachycardia in patients with hypertrophic cardiomyopathy using late gadolinium enhancement and extracellular volume CMR. Eur. Radiol..

[86] Sado D.M., White S.K., Piechnik S.K., Banypersad S.M., Treibel T., Captur G., Fontana M., Maestrini V., Flett A.S., Robson M.D. (2013). Identification and assessment of Anderson-Fabry disease by cardiovascular magnetic resonance noncontrast myocardial T1 mapping. Circ. Cardiovasc. Imaging.

[87] Hinojar R., Varma N., Child N., Goodman B., Jabbour A., Yu C.-Y., Gebker R., Doltra A., Kelle S., Khan S. (2015). T1 mapping in discrimination of hypertrophic phenotypes: Hypertensive heart disease and hypertrophic cardiomyopathy: Findings from the International T1 Multicenter Cardiovascular Magnetic Resonance Study. Circ. Cardiovasc. Imaging.

[88] Gastl M., Gotschy A., von Spiczak J., Polacin M., Bönner F., Gruner C., Kelm M., Ruschitzka F., Alkadhi H., Kozerke S. (2019). Cardiovascular magnetic resonance T2* mapping for structural alterations in hypertrophic cardiomyopathy. Eur. J. Radiol. Open.

[89] Gastl M., Gruner C., Labucay K., Gotschy A., Von Spiczak J., Polacin M., Boenner F., Kelm M., Ruschitzka F., Alkadhi H. (2020). Cardiovascular magnetic resonance T2* mapping for the assessment of cardiovascular events in hypertrophic cardiomyopathy. Open Heart.

